# Comparison of Compound Lidocaine Cream and Lidocaine–Prilocaine Cream for Pain Management in Facial Rejuvenation: A Randomized Controlled Trial

**DOI:** 10.1111/jocd.70865

**Published:** 2026-04-19

**Authors:** Li Liu, Fei Zhang, Cuifang Han, Chen Yuan, Mengqi Han, Changyu Zhang, Li Li

**Affiliations:** ^1^ Plastic Surgery Hospital Chinese Academy of Medical Sciences & Peking Union Medical College Beijing China

**Keywords:** compound lidocaine cream, facial rejuvenation, lidocaine cream, pain management, randomized controlled trial

## Abstract

**Background:**

Effective pain management is essential for enhancing patient satisfaction and treatment compliance during facial rejuvenation procedures. Compound lidocaine cream and lidocaine–prilocaine cream are commonly used topical anesthetics in clinical practice; however, high‐quality randomized controlled trials directly comparing their analgesic efficacy and safety in facial rejuvenation remain limited.

**Objective:**

This study aimed to compare the analgesic efficacy and safety of compound lidocaine cream and lidocaine–prilocaine cream in patients undergoing facial rejuvenation procedures.

**Methods:**

This single‐center, prospective, randomized controlled trial enrolled 100 patients undergoing facial rejuvenation between January 2024 and June 2025. Participants were randomly assigned to receive either compound lidocaine cream (*n* = 47) or lidocaine–prilocaine cream (*n* = 53). The primary outcome was procedural pain intensity, assessed using a 10‐point visual analog scale (VAS). Secondary outcomes included the incidence of adverse reactions, patient satisfaction, and treatment compliance.

**Results:**

Baseline characteristics were comparable between the two groups (*p* > 0.05). The mean pain score was significantly lower in the compound lidocaine cream group than in the lidocaine–prilocaine cream group (2.51 ± 1.80 vs. 3.75 ± 1.74; t = −3.48, *p* < 0.001). Adverse reactions occurred in 4.3% (2/47) of patients in the compound lidocaine cream group and 11.3% (6/53) in the lidocaine–prilocaine cream group, with no statistically significant difference between groups (χ^2^ = 1.89, *p* = 0.169).

**Conclusion:**

Compound lidocaine cream demonstrated superior analgesic efficacy in this clinical comparison; however, differences in anesthetic concentration, application technique, and lack of participant blinding should be considered when interpreting these findings. Larger studies with standardized dosing and blinding are warranted.

## 1 Introduction

With increasing living standards and heightened aesthetic awareness, the demand for facial rejuvenation procedures in medical aesthetics has grown substantially. In 2022, more than 18 million nonsurgical cosmetic procedures were performed worldwide, with facial rejuvenation accounting for a considerable proportion of these treatments [1]. However, many facial rejuvenation procedures are associated with varying degrees of pain, which may adversely affect patients' treatment experience and compliance [2]. Previous studies have demonstrated that pain is a primary concern among patients undergoing aesthetic procedures, and inadequate pain control can reduce both patient satisfaction and willingness to continue treatment [3]. Therefore, effective pain management is essential to enhance procedural safety and improve overall patient satisfaction in facial rejuvenation.

Topical local anesthetics play a crucial role in pain management during facial rejuvenation procedures [4]. Commonly used topical anesthetics in clinical practice primarily include amide‐ and ester‐type agents. Compound lidocaine cream, a representative amide anesthetic, is characterized by rapid onset, strong skin penetration, and a moderate duration of action [5]. Lidocaine–prilocaine cream (EMLA cream) is a eutectic mixture of lidocaine and prilocaine, designed to enhance anesthetic efficacy through the synergistic effects of the two agents.

In recent years, the application of these topical anesthetics in cosmetic and dermatologic procedures has been increasingly investigated [6]. Previous studies have reported that lidocaine–tetracaine formulations provide superior analgesia compared with lidocaine–prilocaine cream during CO_2_ laser treatments, with a high proportion of patients reporting adequate pain relief. Similar findings have been observed in laser‐assisted tattoo removal procedures [7]. However, existing studies have primarily focused on laser‐based treatments, and comparative evidence regarding different facial rejuvenation modalities—such as hydroinjection, gold microneedling, and CO_2_ fractional laser therapy—remains limited [6].

In addition to analgesic efficacy, the safety of topical anesthetics is a major concern in clinical practice. Local anesthetic agents may cause adverse reactions such as erythema, edema, pruritus, and in rare cases, allergic responses [8]. Therefore, evaluation of anesthetic effectiveness must be accompanied by a comprehensive assessment of drug safety. The present randomized controlled trial aimed to compare the analgesic efficacy and safety of compound lidocaine cream and lidocaine–prilocaine cream across different facial rejuvenation procedures, with the goal of providing evidence‐based guidance for the clinical selection of topical anesthetics.

## 2 Methods

### 2.1 Design and Setting

This study was designed as a single‐center, prospective, evaluator‐blinded (rather than double‐blinded), randomized controlled clinical trial and was conducted at a tertiary hospital in Beijing, China, between January 2024 and June 2025. The study protocol was reviewed and approved by the Institutional Review Board of the participating hospital. Written informed consent was obtained from all participants prior to study enrollment.

### 2.2 Participants

Patients presenting for facial rejuvenation at the cosmetic center were screened for eligibility according to predefined criteria. Inclusion criteria were as follows: age between 18 and 65 years; scheduled to undergo facial rejuvenation procedures, including hydro‐injection, gold microneedling, or CO_2_ fractional laser treatment; and the ability to understand and cooperate with pain assessment. Exclusion criteria included a known allergy to amide or ester local anesthetics, pregnancy or lactation, active skin lesions or infections at the treatment site, and concurrent treatments that could affect pain perception. Participant screening and enrollment were performed by attending cosmetic physicians.

### 2.3 Randomization

Randomization was conducted using a computer‐generated random number sequence. Eligible participants were allocated in a 1:1 ratio to either group using a computer‐generated random sequence without block restriction. Minor numerical imbalance between groups was expected under simple randomization and does not indicate failure of allocation. Owing to the distinct physical characteristics of the two topical formulations (e.g., texture, absorption pattern, and occlusion requirement), double blinding was not feasible. Allocation concealment was ensured using sequentially numbered, opaque sealed envelopes prepared by an independent researcher not involved in outcome assessment.

As a result, participants were aware of the treatment they received, while outcome assessors remained blinded to group allocation. Therefore, an evaluator‐blinded design was employed, whereby clinicians responsible for outcome assessment—including pain scores and adverse events—were blinded to treatment allocation throughout the study period. Given that the primary outcome (pain intensity assessed by VAS) was patient‐reported, the lack of participant blinding may introduce potential performance and expectation‐related bias.

### 2.4 Intervention

Two topical anesthetic regimens were evaluated in this study. In the lidocaine–prilocaine cream group, a eutectic mixture containing 2.5% lidocaine and 2.5% prilocaine (EMLA cream) was applied evenly to the entire facial area at a dose of 2–3 tubes (1.5 g per tube), followed by occlusive dressing to enhance transdermal absorption. In the compound lidocaine cream group, 7% lidocaine cream was applied in a comparable amount and allowed to air‐dry without occlusion.

Facial rejuvenation procedures included three commonly used modalities: hydroinjection, in which hyaluronic acid was delivered into the superficial dermis using a multineedle device over 15–20 min; gold microneedling, performed using a radiofrequency device with needle depths ranging from 0.5 to 2.0 mm over 30–45 min; and CO_2_ fractional laser treatment, with energy density adjusted according to clinical requirements during a 20–30‐min session. The application methods followed manufacturer recommendations and routine clinical practice: lidocaine–prilocaine cream is typically applied under occlusion to enhance absorption, whereas the compound lidocaine cream used in this study is commonly applied without occlusion.

### 2.5 Outcomes

The primary outcome was procedural pain intensity, assessed using a 10‐point visual analog scale (VAS), where 0 indicated no pain, 1–3 mild pain, 4–6 moderate pain, and 7–10 severe pain. Secondary outcomes included the incidence of adverse reactions (such as local erythema, edema, pruritus, and allergic reactions), anesthesia onset time (defined as the interval from drug application to onset of effect), anesthesia duration (from onset to resolution of effect), patient satisfaction measured using a 5‐point Likert scale, and treatment compliance, defined as the patient's willingness to undergo subsequent treatments.

### 2.6 Sample Size

The sample size was calculated a priori using G*Power software (version 3.1). Based on previous randomized controlled trials comparing the analgesic efficacy of lidocaine–tetracaine cream and lidocaine–prilocaine cream in dermatologic laser procedures, an effect size (Cohen's d) of 0.7 was assumed. With a two‐sided significance level (α) of 0.05 and a statistical power (1−β) of 80%, a minimum of 33 participants per group was required. To account for an anticipated dropout rate of approximately 20%, a total sample size of 100 patients was planned.

### 2.7 Statistical Analysis

Statistical analyses were performed using SPSS software (version 25.0). Continuous variables were expressed as mean ± standard deviation and compared between groups using independent‐samples *t*‐tests or the Mann–Whitney U test, as appropriate. Categorical variables were presented as frequencies and percentages and analyzed using the chi‐square test or Fisher's exact test. A two‐sided *p* value < 0.05 was considered statistically significant.

## 3 Results

### 3.1 Baseline Characteristics of the Patients

A total of 100 patients receiving facial rejuvenation treatment were enrolled in this study, including 47 patients in the compound lidocaine cream group and 53 patients in the lidocaine–prilocaine cream group. There were no statistically significant differences in baseline characteristics such as age, gender, skin type, and treatment items between the two groups (*p* > 0.05), which were well comparable (Table 1).

**TABLE 1:** Comparison of baseline characteristics of patients.

Characteristics	Compound lidocaine cream group (*n* = 47)	Lidocaine cream group (*n* = 53)	*p*
Age (years, x̄±s)	35.2 ± 8.6	33.8 ± 9.1	0.423
Gender (female/male)	42/5	48/5	0.892
Skin type (type III/IV)	35/12	38/15	0.756
Treatment items			0.834
Water light injection	18	21	
Gold microneedles	16	21	
CO_2_ fractional laser	13	11	

### 3.2 Main Outcome Measure: Pain Score

#### 3.2.1 Overall Pain Score Comparison

A significant between‐group difference was observed in the primary outcome of procedural pain (Table 2). Patients in the compound lidocaine cream group reported significantly lower mean pain scores than those in the lidocaine–prilocaine cream group (2.51 ± 1.80 vs. 3.75 ± 1.74). This difference was statistically significant based on both the independent‐samples *t*‐test (*t* = −3.48, *p* < 0.001) and the Mann–Whitney U test (*U* = 796.0, *p* = 0.002). The calculated effect size (Cohen's d = −0.70) indicated a moderate clinically meaningful difference between groups.

**TABLE 2:** Comparison of overall pain scores between the two groups.

Variable	Compound lidocaine cream group (*n* = 47)	Lidocaine–prilocaine cream group (*n* = 53)	Statistic	*p*
Pain score (continuous data)				
Mean ± standard deviation	2.51 ± 1.80	3.75 ± 1.74	*t* = −3.48	< 0.001*
Median (interquartile range)	2.0 (1.0–4.0)	3.0 (2.0–5.0)	*U* = 796.0	0.002*
Pain severity level [*n* (%)]			*χ* ^2^ = 12.45	0.006*
No pain (0)	12 (25.5)	5 (9.4)		
Mild pain (1–3)	25 (53.2)	20 (37.7)		
Moderate pain (4–6)	9 (19.1)	23 (43.4)		
Severe pain (7–10)	1 (2.1)	5 (9.4)		

*Note:* *p < 0.05 indicates statistical significance.

Further analysis of pain severity categories also demonstrated a significant between‐group difference (*χ* ^2^ = 12.45, *p* = 0.006). A higher proportion of patients in the compound lidocaine cream group reported no pain or mild pain, whereas moderate to severe pain was more frequently reported in the lidocaine–prilocaine cream group.

#### 3.2.2 Pain Ratings for Different Treatment Items

Stratified analysis by treatment modality demonstrated that compound lidocaine cream consistently provided superior analgesia across all facial rejuvenation procedures (Table 3). During hydroinjection, the mean pain score in the compound lidocaine cream group was significantly lower than that in the lidocaine–prilocaine cream group (1.44 ± 1.15 vs. 2.48 ± 1.31; *t* = −2.67, *p* = 0.011). Similarly, in gold micro‐needling treatments, patients receiving compound lidocaine cream reported significantly lower pain scores compared with those receiving lidocaine–prilocaine cream (2.94 ± 1.77 vs. 4.19 ± 1.89; *t* = −2.18, *p* = 0.034). In CO_2_ fractional laser procedures, the compound lidocaine cream group also experienced significantly less pain (4.15 ± 1.68 vs. 5.64 ± 1.81; *t* = −2.45, *p* = 0.020).

**TABLE 3:** Pain scores for different treatment items.

Treatment items	Compound lidocaine cream group	Lidocaine–prilocaine cream	T‐score	*p*
Water light injection	1.44 ± 1.15	2.48 ± 1.31	−2.67	0.011 *
Gold microneedles	2.94 ± 1.77	4.19 ± 1.89	−2.18	0.034 *
CO_2_ fractional laser	4.15 ± 1.68	5.64 ± 1.81	−2.45	0.020 *

*Note:* *p < 0.05 indicates statistical significance.

### 3.3 Secondary Outcomes

#### 3.3.1 Adverse Effects

The incidence of adverse reactions was low in both groups. All observed adverse events were mild local erythema, which resolved spontaneously or with minimal intervention. The adverse reaction rate was 4.3% (2/47) in the compound lidocaine cream group and 11.3% (6/53) in the Lidocaine–prilocaine cream group. This difference was not statistically significant (χ^2^ = 1.89, *p* = 0.169), as detailed in Table 4.

**TABLE 4:** Incidence of adverse reactions of the two drugs.

Adverse reactions	Compound lidocaine cream group (*n* = 47)	Lidocaine–prilocaine cream (*n* = 53)	*p*
Localized erythema	2 (4.3%)	6 (11.3%)	0.169
Edema	0 (0%)	0 (0%)	—
Itching	0 (0%)	0 (0%)	—
Allergic reactions	0 (0%)	0 (0%)	—
Total	2 (4.3%)	6 (11.3%)	0.169

#### 3.3.2 Anesthesia Profile

No significant differences were observed in the anesthesia profiles of the two topical anesthetics. The mean onset time was comparable between the compound lidocaine cream group and the lidocaine–prilocaine cream group (28.5 ± 5.2 min vs. 30.1 ± 6.1 min; *t* = −1.39, *p* = 0.168). Similarly, the duration of anesthesia did not differ significantly between groups (45.2 ± 8.6 min vs. 42.8 ± 9.1 min; *t* = 1.34, *p* = 0.183).

#### 3.3.3 Patient‐Reported Outcomes and Compliance

Treatment with compound lidocaine cream led to significantly higher patient satisfaction scores (4.6 ± 0.5) compared to lidocaine–prilocaine cream (4.1 ± 0.8; *t* = 3.67, *p* < 0.001). Consequently, patients in the compound lidocaine cream group demonstrated significantly higher treatment compliance, with 95.7% (45/47) willing to receive subsequent treatments, compared to 83.0% (44/53) in the control group (χ^2^ = 4.12, *p* = 0.042).

## 4 Discussion

In this randomized controlled trial, we compared the analgesic efficacy and safety of compound lidocaine cream and lidocaine–prilocaine cream in patients undergoing facial rejuvenation procedures. The results demonstrated that compound lidocaine cream provided significantly superior pain relief compared with lidocaine–prilocaine cream while maintaining a comparable safety profile. These findings offer clinically relevant evidence to support the selection of topical anesthetic agents in facial rejuvenation practice.

With respect to pain control, patients treated with compound lidocaine cream reported significantly lower pain scores than those receiving lidocaine–prilocaine cream, indicating superior analgesic efficacy during facial rejuvenation procedures. This advantage was consistently observed across different treatment modalities, including hydro‐injection, gold microneedling, and CO_2_ fractional laser therapy [9]. It is well established that topical anesthetic efficacy is influenced by multiple factors, including drug concentration, molecular properties, vehicle composition, and application conditions [10]. Because the present study did not control for total anesthetic dose between groups, the independent contribution of formulation characteristics versus concentration cannot be definitively determined. As such, the findings of this study demonstrate comparative clinical effectiveness between the two commercially available preparations, rather than mechanistic superiority of one formulation over the other. Future randomized trials standardizing total anesthetic concentration would be required to isolate formulation‐specific effects.

Regarding safety, no significant difference in the incidence of adverse reactions was observed between the two groups, and all reported events were mild, localized erythema that resolved without the need for specific intervention. No systemic adverse reactions were detected, which may be attributed to the topical application on intact facial skin and strict control of dosage within established safety limits [11]. The occurrence of adverse reactions is influenced not only by drug composition and concentration but also by anatomical and physiological characteristics of the application site, such as skin thickness, stratum corneum integrity, and local vascular distribution. Compared with mucosal tissues, facial skin provides a more effective barrier to systemic absorption, thereby reducing the risk of systemic toxicity. Compared with mucosal tissue, facial skin has a better barrier function, which effectively limits the systemic absorption of drugs, thereby reducing the related systemic risks [12]. In this study, no severe skin reactions were observed in either group, indicating that both local anesthetics are relatively safe for short‐term use in facial rejuvenation.

Nevertheless, caution is warranted when interpreting the safety findings. The present study was primarily powered to detect differences in pain intensity rather than adverse event incidence. With a total sample size of 100 participants, the study may have been underpowered to identify clinically meaningful differences in safety outcomes. Although no statistically significant difference in adverse events was observed, the numerically higher incidence in the lidocaine–prilocaine group (11.3% vs. 4.3%) suggests that potential safety differences cannot be definitively excluded. Larger studies specifically designed to assess safety endpoints are needed for more robust comparisons.

Patient satisfaction and treatment compliance are also important indicators to evaluate the effectiveness of pain management. Patient satisfaction scores were significantly higher in the compound lidocaine cream group than in the Lidocaine–prilocaine cream group, and a higher proportion of patients were willing to accept subsequent treatment. Good pain management not only reduces the discomfort of patients but also improves the overall treatment experience. On the one hand, effective analgesia can reduce the anxiety and tension of patients during the treatment. On the other hand, a comfortable treatment experience helps to build patients' sense of trust in doctors, thereby improving treatment compliance. These factors are particularly important in the field of cosmetic and plastic surgery because most of these treatments are elective procedures, and the patient's psychological state and subjective experience often directly affect the treatment decision. The results of this study are similar to a study by Huang et [13]. This suggests that optimizing pain management is a key factor in improving overall patient satisfaction, both in invasive and noninvasive cosmetic treatments. In addition, high treatment compliance means that patients are more likely to complete the prespecified treatment plan on time and will not give up halfway due to pain concerns, which is particularly critical for facial rejuvenation procedures that require multiple treatments, such as serial laser treatments or regular water light injections. Therefore, in terms of long‐term treatment success and patient retention, investing in more effective topical anesthetic agents may lead to better clinical outcomes and patient loyalty for healthcare institutions. This further confirms the advantages of compound lidocaine cream in enhancing the patient's treatment experience.

In conclusion, this randomized controlled trial demonstrated that compound lidocaine cream provides significantly superior analgesic efficacy compared with lidocaine–prilocaine cream during facial rejuvenation procedures, while maintaining a comparable safety profile. This advantage was consistently observed across different treatment modalities, including hydro‐injection, gold microneedling, and CO_2_ fractional laser therapy. Patients treated with compound lidocaine cream experienced lower pain intensity, higher satisfaction, and improved treatment compliance.

Based on these findings, compound lidocaine cream demonstrated superior analgesic efficacy in this study; however, because the total anesthetic concentration differed between formulations, further studies controlling for anesthetic dose are needed before definitive conclusions regarding formulation superiority can be drawn. Furthermore, the randomized design and standardized pain assessment employed in this study provide methodological insight for future research on pain management in aesthetic medicine. Further investigations involving diverse patient populations, skin types, and pharmacoeconomic evaluations are warranted to support broader clinical decision‐making. Additionally, subgroup analyses according to procedure type were exploratory and involved relatively small sample sizes within each modality; therefore, these findings may be underpowered and should be interpreted cautiously.

## Author Contributions

Li Liu and Fei Zhang contributed equally to the study design, data collection, and manuscript drafting. Cuifang Han, Chen Yuan, and Mengqi Han were responsible for patient recruitment and clinical data acquisition. Changyu Zhang performed the statistical analysis and interpretation of data. Li Li conceptualized the study, supervised the research process, and critically revised the manuscript. All authors read and approved the final manuscript.

## Disclosure

The authors have nothing to report.

## Ethics Statement

This study was conducted in accordance with the Declaration of Helsinki. The study protocol was approved by the Institutional Review Board of the Plastic Surgery Hospital, Chinese Academy of Medical Sciences & Peking Union Medical College.

## Consent

Written informed consent was obtained from all participants prior to enrollment.

## Conflicts of Interest

The authors declare no conflicts of interest.

## Data Availability

Research data are not shared.
